# Recyclable, Self‐Healing Solid Polymer Electrolytes by Soy Protein‐Based Dynamic Network

**DOI:** 10.1002/advs.202103623

**Published:** 2022-02-10

**Authors:** Weidong Gu, Feng Li, Tao Liu, Shanshan Gong, Qiang Gao, Jianzhang Li, Zhen Fang

**Affiliations:** ^1^ MOE Key Laboratory of Wood Material Science and Application & Beijing Key Laboratory of Wood Science and Engineering Beijing Forestry University Beijing 100083 China; ^2^ Department of Biochemistry & Molecular Biology Great Lakes Bioenergy Research Center‐Michigan State University East Lansing MI 48824 USA

**Keywords:** solid polymer electrolytes, soy protein isolate, vitrimers

## Abstract

Compared to traditional organic liquid electrolytes, which often present leakage, flammability, and chemical stability problems, solid polymer electrolytes (SPEs) are widely regarded as one of the most promising candidates for the development of safer lithium‐ion batteries. Vitrimers are a new class of polymer materials consisting of dynamic covalent networks that can change their topology by thermally activated bond‐exchange reactions. Herein, the recyclable and self‐healing solid polymer electrolytes (SPEs) with a soy protein isolate (SPI)‐based imine bond dynamic network are reported. This malleable covalent cross‐linked network polymer can be reshaped and recycled at high temperature (100 °C) or only with water at ambient temperature (25 °C), which may realize the green processing of energy materials. The introduction of bis(trifluoromethane) sulfonimide lithium (LiTFSI) significantly reinforces the conductivity of the dynamic network to a maximum of 3.3 × 10^−4^ S cm^‐1^. This simple and applicable method establishes new principles for designing scalable and flexible strategies for fabricating polymer electrolytes.

## Graphical abstract

The recyclable and self‐healing solid polymer electrolytes (SPEs) with a soy protein isolate (SPI)‐based imine bond dynamic network display favorable mechanical strength (77.4 MPa), high ionic conductivity (3.3 × 10^−4^ S cm^−1^). Thus, the SPEs based on the imine bond dynamic network of SPI is a promising platform for recyclable and repairable electrolytes.

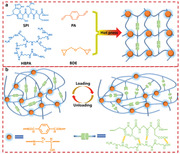

## 1 Introduction

Lithium‐ion batteries (LIB) have been widely used in daily life, in which their cycle life, safety, and system stability are crucial to meet the increasing demand.^[^ ^1^ ^]^ The electrolyte of LIB can be categorized into liquid electrolyte and solid electrolyte, in which the technology of liquid electrolyte is relatively mature.^[^ ^2^, ^3^ ^]^ However, its insurmountable disadvantages, including the utilization of flammable volatile organic electrolyte solvents and electrolyte leakage limited its practical application.^[^ ^4^ ^]^ In contrast, solid electrolytes are capable of inhibiting the formation/growth of lithium dendrites, incombustibility, and a large electrochemical voltage window, showing great potential as a substitute for liquid electrolytes.^[^ ^5^ ^]^ Among numerous solid electrolytes, solid polymer electrolytes (SPEs) have outstanding advantages over inorganic ceramic electrolytes, including high flexibility, easy processability, and low interface resistance. For instance, poly (ethylene oxide) (PEO) or polyacrylonitrile‐based matrices used commonly available LiClO_4_ or bis(trifluoromethane) sulfonimide lithium (LiTFSI),^[^ ^3^, ^6^ ^]^ but the resulting products still cannot satisfy high ionic conductivity at room temperature, mechanical stability and high‐temperature stability, and chemical inertness during battery cycling.^[^ ^7^, ^8^, ^9^ ^]^ An effective way to improve the conductivity of SPEs is to increase the mobility of lithium ions by inhibiting the crystallization of polymer chains and simultaneously increasing the mobility of polymer chains.^[^ ^10^ ^]^ Generally, the employment of electrolytes comprising a covalent bond cross‐linking matrix is considered as an effective strategy to improve the mechanical properties, thermal stability and inhibit dendrite growth of SPEs.^[^ ^11^, ^12^ ^]^ While the shortcoming of these electrolytes containing permanent covalent bond cross‐linked network is also significant, that is, cross‐linked network cannot be processed after curing, nor can it be restored to use after battery failure. Therefore, it is highly desirable to formulate SPEs that can be processible after cross‐linking and solidification or repairable after damage.

The application of self‐healing materials attracted wide attention in various areas due to their unique merits, such as ion conductors,^[^ ^12^ ^]^ supercapacitors, and electronic skins.^[^ ^13^ ^]^ However, most of the self‐healing materials currently used for electrolytes are constructed with relatively weak noncovalent crosslinking networks, showing poor mechanical properties and low creep resistance. The introduction of exchangeable chemical bonds provides an attractive chemical strategy to introduce plasticity in the crosslinked polymer network, leading to dynamic cross‐linking.^[^ ^14^ ^]^ Covalently adaptable networks (CANs) are covalently cross‐linked polymers that behave similarly to classic thermosetting materials at room temperature and can be cross‐linked and/or chain exchanged at elevated temperatures with ductility and re‐processability.^[^ ^15^ ^]^ Vitrimer, a new class of associative CANs was synthesized by Leibler et al. for the first time,^[^ ^16^ ^]^ and its dynamic chemistries have been extensively explored, including transesterification,^[^ ^17^ ^]^ boronic ester exchange,^[^ ^18^ ^]^ imine metathesis,^[^ ^19^, ^20^ ^]^ and disulfide metathesis.^[^ ^21^ ^]^ On the contrary, to the best of our knowledge, limited studies of vitrimer in the application in lithium transmission were reported.

Herein, we report a novel soy protein isolate (SPI) based matrix containing a dynamic imine cross‐linked network, in which the addition of LiTFSI endowed the resultant material with a conductivity of more than 3.3 × 10^−4^ S cm^‐1^ (at 30 °C). This transparent and uniform film was prepared by the solvent casting method with high inorganic material loading (the mass ratio of SPI and LiTFSI is 1:4). The dynamic imine exchange reaction of the self‐healable polyelectrolyte enabled rapid structure or function recovery and provided effective shape adaptability or configurability. Overall, the dynamic SPEs are promising feedstocks for recyclable and repairable electrolytes.

## 2 Result and Discussion

### 2.1 Physicochemical Properties of SPI‐Based Vitrimer

**Figure 1:**
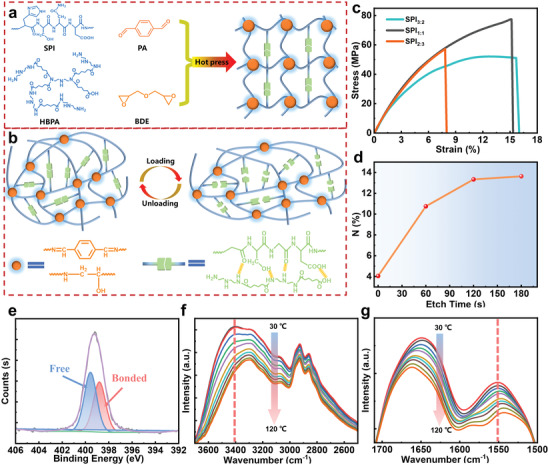
a) Preparation of the dynamic covalent networks of SPI‐based vitrimer. b) Schematic illustration of the energy dissipation mechanism through reversible breaking and reformation of hydrogen bonds under deformation. c) The stress–strain curve of SPI‐based vitrimers. d) The atomic percentage of N as a function of etching time by wide‐scan XPS provides (the etching time is proportional to etching depth on the sample surface). e) Narrow‐scan XPS spectra of N 1s orbital and their fitting curves indicate the presence of both bonded N–H (red) and free N–H (blue) groups. f,g) Temperature‐dependent FTIR spectra of SPI_1:1_ upon heating from 30 to 120 °C at 2600 to 3600 cm^−1^, and 1500 to 1700 cm^−1^, respectively.

The recyclable and self‐healable SPI‐based vitrimer with dynamically cross‐linked polyimine network was prepared by using commercially available starting materials SPI and hyperbranched polyamide (HBPA), in which, HBPA was synthesized from adipic acid and diethylenetriamine in a simple one‐step process (Figure S1, Supporting Information). Briefly, the rigid SPI and flexible HBPA segments were cross‐linked by 1,4‐phthalaldehyde (PA) and 1,4‐butanediol diglycidyl ether (BDE) (**Figure** **1**a). After curing, disappearance of the stretching vibration peaks corresponding to the aldehyde group (1684 cm^−1^) and the alkylene oxide ring (908 cm^−1^) in the FTIR and the appearance of peak associate with the imine bond (1634 cm^−1^) (Figure S2, Supporting Information) validated the completed crosslinking reactions, indicating the imine bond has been integrated into the network.

In this context, *x–y* in the sample code of SPI*_x_*_–_ *_y_* refers to the molar ratio of PA:BDE under SPI/HBPA mass ratio is 1:1. As shown in Figure 1c (Figure S3, Supporting Information), when the molar ratio of PA/BDE is 1:1 (SPI_2_, SPI_1:1_), the SPI‐based vitrimer (SPI_1:1_) presented the highest tensile strength (77.4 MPa) and modulus (1.12 GPa). The cross‐linked network can change its topological structure and release stress through imine exchange reaction at high temperatures, thereby endowing the material reworkability. Meanwhile, functional groups such as hydroxyl, amino, and amide groups in the cross‐linked network can form hydrogen bonds and serve as sacrificial bonds, which greatly improved the mechanical properties while maintaining the dynamic properties of the resulting SPI‐based vitrimer. Figure 1b depicts the formation of the interface hydrogen bond between the SPI molecular chain and HBPA. The atomic percentage of N is provided by the amino group, as the etching depth in the sample increased and stabilized after 120 s, as confirmed by the wide scan XPS (Figure 1d and Figure S4, Supporting Information). The bimodal composition of the N 1s narrow scan XPS spectrum (Figure 1e and Figure S5, Supporting Information) confirms the presence of bound N–H and free N–H groups (bound N–H groups have lower binding energy than free N–H groups).^[^ ^22^ ^]^ According to their temperature‐sensitive characteristics, temperature‐dependent infrared spectroscopy provided evidence of the existence of supermolecular hydrogen bonds at the interface, as shown in Figure 1f,g (Figure S6, Supporting Information).^[^ ^23^, ^24^ ^]^ When the temperature raised from 30 °C to 120 °C, the intensities of the amino stretching vibrations at 3392.8 and 1550.5 cm^−1^ declined with increasing temperature and gradually shifted to a low wavenumber of 3296.9 and 1543.1 cm^−1^, respectively. These spectral changes indicate the breakage of the hydrogen bonds under evaluated temperatures, accompanied with the disassociation of the hydrogen bonds —OH and —NH groups into “free” groups.^[^ ^23^ ^]^

**Figure 2:**
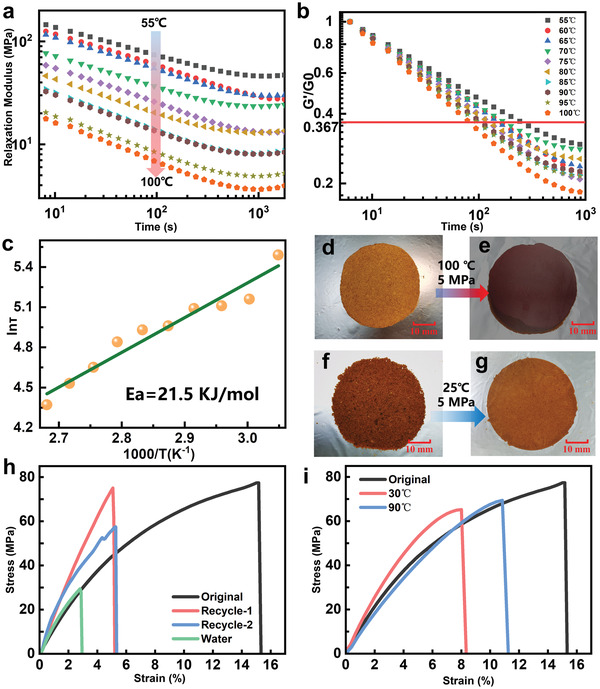
a) The stress relaxation curves of the SPI_1:1_ vitrimer film at different temperatures over a time course of 30 min. b) Normalized stress relaxation curves of the SPI_1:1_ vitrimer film at various temperatures. c) Fitting of the relaxation times to the Arrhenius equation. d) SPI_1:1_ vitrimer powder obtained by grinding the SPI_1:1_ vitrimer film. e) SPI_1:1_ vitrimer film formed from powder by hot‐pressing at 100 °C and 5 MPa for 10 min. f,g) SPI_1:1_ vitrimer film formed from wet powder, which the weight ratio of dry powder: water is 2:1, by pressing at 25 °C and 5 MPa for 24 h. h) The stress–strain curves for the film through two generations of recycling from powder to film under hot press and the stress–strain curves of the film, which formed from wet powder under cold press. i) The strain–stress curves of dry SPI_1:1_ vitrimer film after immersed in warm water (30 °C) and hot water (90 °C).

The dynamic adaptive characteristics of the SPI_1:1_ (the mass ratio of SPI/HBPA is 1:1 and the molar ratio of PA/BDE is 1:1) vitrimer film were evaluated by testing the relaxation modulus as a function of time and temperature. **Figure** **2**a depicts the results of a series of relaxation tests in a wide temperature range (55–100 °C), in which the normalized stress relaxation modulus gradually decreased with time at different temperatures. Specifically, the dynamic imine bond exchange reaction was initiated,^[^ ^25^ ^]^ and the normalized relaxation modulus dropped from 1 to 0.2 after 30 min (Figure 2b). Based on the Maxwell's model of viscoelastic fluid, the relaxation time (*τ*) is determined as the time required to relax to 1/e (36.7%) of the initial modulus,^[^ ^20^, ^26^ ^]^ which declines sharply with the increase of temperature. The relationship between stress relaxation time and 1000/*T* was obtained by fitting the Arrhenius’ law in Figure 2c (Equation S1, Supporting Information), which afforded a relaxation activation energy (*E*_a_) of 21.5 KJ mol^−1^. *E*_a_ reflects the activation energy of the imine exchange reaction, indicating that large *τ* at low temperature provides necessary dimensional stability, while small *τ* at high temperature provides rapid response to shape editing and self‐healing.^[^ ^13^ ^]^

Successful determination of the plasticity of the SPI_1:1_ vitrimer film stimulated us to explore the recyclability of the material by studying its durability with dynamic imine bonds toward the complete reprocessing from powder to a film. The synthesized polymer powder was molded at 100 °C under 5 MPa pressure for 30 min (Figure 2d). After cooling to room temperature, the formed dark brown film (Figure 2e), was ground into fine powder and repeated for two additional times. After two generations of cycles, as shown in Figure 2h, the regenerated materials showed high mechanical strength, indicating the impressive recyclability of the catalyst‐free dynamic reaction of imine. SPI and HBPA are known to easily absorb water, and imine bonds are also susceptible to hydrolyze and cleave. The characteristic stress–strain behavior under different atmospheric humidity (Figure S7, Supporting Information) revealed the improved flexibility of the SPI1:1 vitrimer film with increasing atmospheric humidity. Notably, the SPI_1:1_ vitrimer film maintained a high strength (32 MPa) under 50% humidity meanwhile exhibited an elongation at break of more than 250%, showing unparalleled toughness (67.64 MJ m^‐3^). When the SPI_1:1_ vitrimer film was immersed in warm water (30 °C) and hot water (90 °C), the hard glass‐like SPI_1:1_ vitrimer film became soft and elastic as it expanded. After removal from water and dried (Figure S8, Supporting Information), the SPI1:1 vitrimer film still maintained a high level (Figure 2i). Addition of water in the synthesized SPI_1:1_ vitrimer powder (power: water = 2:1, w/w) afforded a transparent SPI_1:1_ vitrimer film by simple molding at room temperature under 5 MPa for 24 h (Figure 2f,g). The resulting SPI_1:1_ vitrimer film exhibited typical hard polymer characteristic after removal of the moisture, that is, high elastic modulus and low elongation at break, though the strength was lower than that of the SPI_1:1_ vitrimer film repaired with hot‐pressed powder (Figure 2h). When the dry powder was reprocessed under the same conditions, a brittle compacted powder SPI_1:1_ vitrimer block was obtained (Figure S9, Supporting Information), illustrating that in the absence of water at room temperature, there is no macroscopic evidence of the occurring of the imine bond exchange reaction. Usage of water to reshape thermosetting polymers is both environmental and economical friendly, of particular interest, the green and room temperature processing conditions are feasible for this class of important functional polymers.

### 2.2 Electrochemical Performance of SPI‐Based Vitrimer Electrolyte

**Figure 3:**
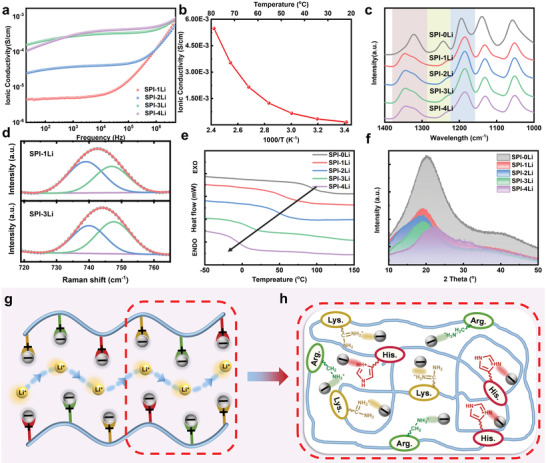
a) The ionic conductivity of SPI‐Li based SPEs with different proportions of LiTFSI. b) The ionic conductivity of the SPI‐3Li under different temperature. c) FTIR (1000 to 1400 cm^−1^) of SPI‐Li based SPEs with different proportions of LiTFSI. d) The Raman spectra (720 to 760 cm^−1^) of SPI‐1Li and SPI‐3Li. e,f) The DSC curves and XRD patterns of SPI‐Li based SPEs with different proportions of LiTFSI, respectively. g) Schematic diagram of the Li^+^ transport process. h) SPI locks anions in the protein chain through positively charged amino acids, which promotes the jumping of Li^+^.

The ion conductivity of the SPI_1:1_ vitrimer film with different g LiTFSI loading was measured by electrochemical impedance spectroscopy (EIS) to obtain insights about ion transport. To optimize the ratio of SPI to LiTFSI in the SPEs, different LiTFSI contents were selected using a mass ratio of SPI/ LiTFSI of 1:1, 1:2, 1:3, and 1:4, respectively. In this context, the sample code of SPI‐zLi refers to the mass radio of SPI and LiTFSI. **Figure** **3**a shows the conductivity (*σ*) as a function of frequency (*ω*), with the plateau value as the direct current conductivity.^[^ ^12^ ^]^ With the increase of the amount of LiTFSI loading, the conductivity of the SPI‐based SPEs gradually increased. The SPI‐3Li (the mass ratio of SPI/HBPA/LiTFSI is 1:1:3 and the molar ratio of PA/BDE is 1:1) exhibited the highest conductivity (3.3 × 10^−4^ S cm^‐1^, at 30 °C). The conductivity increased with temperature (Figure 3b and Figures S10 and S11, Supporting Information), and displayed a non‐Arrhenius dependence on temperature. SPI is a natural polymer constituted by 18 different amino acids (see Table S2, Supporting Information) covalently linked by peptide bonds,^[^ ^27^, ^28^ ^]^ and positively charged amino acids (such as arginine, lysine, and histidine) can capture anions.^[^ ^27^, ^29^ ^]^ As shown in the transport mechanism Figure 3g,h, the CF_3_SO_3_^−^ is immobilized and firmly locked in the protein by specific positively charged side groups, which benefited to inhibit the concentration polarization of anions and promote the migration of Li^+^.^[^ ^30^ ^]^

FTIR and Raman spectroscopy were used to identify the anion trapping effect of SPI. Figure 3c shows the detailed FTIR spectra of LiTFSI and SPI‐Li film in the range of 1000–1400 cm^−1^. Obviously, the —CF_3_ stretching peak of LiTFSI shifted from 1194 to 1185 cm^−1^, while the —SO_2_ stretching peak at 1321 cm^−1^ moved to 1346 cm^−1^.^[^ ^31^ ^]^ In addition, the —NH_2_ peak shifted from 3274 to 3370 cm^−1^ (Figure S12, Supporting Information). These peak shifts indicated the interaction between SPI and CF_3_SO_3_^−^ which ensured the solvation of LiTFSI and transport of Li^+^.^[^ ^32^, ^33^ ^]^ Raman spectroscopy further proved that the interaction between CF_3_SO_3_^−^ and SPI, (Figure 3d), in which the peaks at 740 and 747 cm^−1^ corresponding to dissociated ions and ion pairs, respectively, indicating the presence of free lithium ions in the polymer system.^[^ ^31^, ^32^, ^34^ ^]^ DSC measurements suggested that addition of LiTFSI significantly lowered the glass transition temperature (*T*_g_) of SPI‐Li (Figure 3e, attributing to the improved mobility of polymer segments, which promoted the movement of Li^+^).^[^ ^35^ ^]^ The circular dichroism (CD) measurements (Figure S13, Supporting Information) revealed that soy protein displays a typical *β*‐sheet conformation with an absorption 208 nm. After adding LiTFSI, the CD intensities at 208 nm decreased by 5 mdeg likely due to the interaction between LiTFSI and soy protein, resulting in a significant disruption of the soy protein *β*‐sheet conformation. We also explored the crystalline phase of each component in the composite electrolyte through XRD testing (Figure 3f). The diffraction peak of LiTFSI vanished in the electrolyte, indicating that LiTFSI completely dissolved in the SPI matrix. It is notable that in the composite electrolyte system, with the increase in the amount of LiTFSI, the SPI peak intensity apparently dropped (Figure 3f), implying that the transition of SPI‐3Li from crystal phase to amorphous phase is conducive to the migration of Li^+^ in the unconstrained polymer chain.^[^ ^36^ ^]^

**Figure 4:**
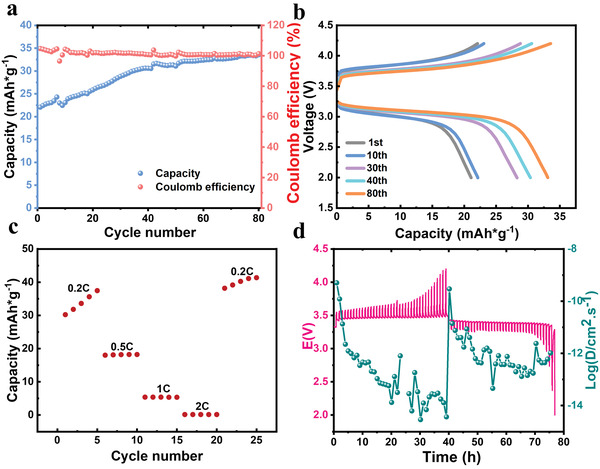
a) Galvanostatic cycling performances of Li|SPI‐3Li|LiFePO_4_ cells at 0.2 C (60 °C). b) Charge/discharge profiles of SPI‐3Li electrolytes. c) Rate capability of Li|SPI‐3Li|LiFePO_4_ cells at 60 °C. d) GITT profiles at the discharge/charge process (red curve), diffusion coefficients calculated from GITT profiles (green curve).

In this study, the electrochemical tests were carried out using CR2025‐type coin cells. The rate performance and cycle performance of coin half‐cell were tested at 60 °C. As displayed in **Figure** **4**a, the cells function normally and stably at 0.2 C, 0.5 C, 1 C, 4 C, 0.2 C current density. The discharge specific capacity after five cycling under different current densities were 33.6, 18.1, 5.3, 0.15 mA h g^−1^, respectively. When the current density returns to 0.2 C, its discharge specific capacity can still quickly recover to 39.1 mA h g^−1^. The specific discharge capacity can maintain at 32.6 mA h g^−1^, with average coulombic efficiency close to 100% during the cycle, suggesting its excellent cycle stability (Figure 4b,c). The galvanostatic intermittent titration (GITT) can be used to calculate the kinetics of Li^+^ diffusion coefficient (*D*_Li+_). Figure 4d (red curve) displays the voltage response of SP‐3Li electrodes during the charge/discharge process. The minor change in potential during each relaxation process corresponds to the overpotential during the corresponding charge/discharge process. As shown in Figure 4d (green curve), the SP‐3Li electrolyte has a high *D*_Li+_ value during the charge/discharge process, which exhibits excellent Li^+^ diffusion kinetics, evidenced by the presence of more electrochemically active sites.

### 2.3 The Stability of SPI‐Based Vitrimer Electrolyte

**Figure 5:**
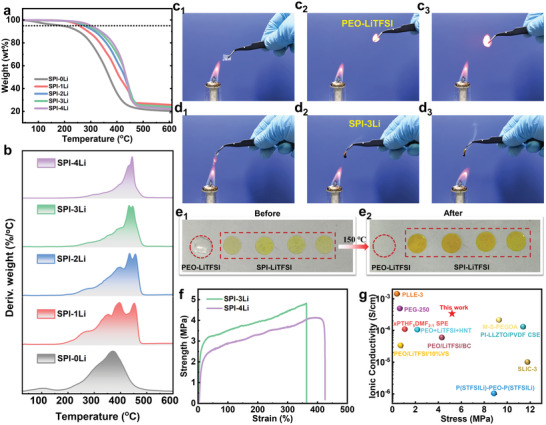
a,b) Thermal stability and of SPI‐Li based SPEs with different proportions of LiTFSI. c,d) Photo images of flame test on a regular PEO/LiTFSI film and SPI‐3Li film. e) Photo images of PEO/LiTFSI film and SPI‐Li based SPEs film before/after exposure to thermal shock (150 °C, 0.5 h), respectively. f) Mechanical properties of SPI‐Li based SPEs with different proportions of LiTFSI. g) Comparison of the ionic conductivity and stress of SPI‐3Li to other polymer electrolytes reported in the literature. (Poly(styrene trifluoromethanesulphonylimide of lithium)‐poly(ethylene oxide)‐poly(styrene trifluoromethanesulphonylimide of lithium) (P(STFSILi)‐PEO‐P(STFSILi)),^[^ ^38^ ^]^ poly(ethylene oxide)‐lithium bis(trifluoromethanesulfonyl)‐10%vermiculite sheets (PEO/LiTFSI/10%VS),^[^ ^9^ ^]^ P(EO)_15_LiTFSI‐0.2Li_7_La_3_Zr_2_O_12_‐1.1ethylene carbonate (PLLE‐3),^[^ ^36^ ^]^ PEO+LiTFSI(EO:Li = 15:1)+halloysite nanotube(10%) (HNT),^[^ ^39^ ^]^ metal‐organic frameworks‐tetrakis (3‐mercaptopropionic acid) pentaerythritol‐poly(ethylene glycol) diacrylate (M‐S‐PEGDA),^[^ ^40^ ^]^ crosslinked poly(tetrahydrofuran)‐N,N‐dimethylformamide solid polymer electrolytes (xPTHF5DMF2:1 SPE),^[^ ^41^ ^]^ poly(ethylene glycol)‐polyhedral oligomeric silsesquio‐xanes(PEG250),^[^ ^45^ ^]^ PEO/LiTFSI/bacterial cellulose(PEO/LiTFSI/BC),^[^ ^42^ ^]^ porous polyimide‐Li_6.75_La_3_Zr_1.75_Ta_0.25_O_12_‐poly(vinylidene fluoride) (PI‐LLZTO/ PVDF),^[^ ^43^ ^]^ supramolecular lithium ion conductor (SLIC).^[^ ^44^ ^]^)

Thermal stability is an important indicator of the safety of the electrolyte applications;^[^ ^8^, ^37^ ^]^ thus, thermogravimetric analysis (TGA) was performed (**Figure** **5**a,b). Obviously, employment of LiTFSI improved the initial degradation temperature of SPI‐Li based SPEs from 200 °C to 240 °C. The degradation of SPI‐3Li electrolyte mainly occurred at 240–480 °C, indicating its high thermal stability, which is ideal for use as an electrolyte. The flame‐retardant properties of SPI‐Li‐based SPEs film were studied by flammability tests, in which a PEO/LiTFSI film was used as the reference sample. In the flammability tests, the SPI‐3Li film showed good flame retardancy. As shown in Figure 5c, PEO/LiTFSI immediately caught fire and burned violently as soon as contacting with fire. In sharp contrast, the SPI‐3Li film was distorted due to high temperature without causing fire even when it was in contact with the flame for a few seconds (Figure 5d). Under thermal shock conditions (150 °C, 0.5 h), the PEO/LiTFSI film melted, while the size and morphology of SPI‐3Li film did not substantial change (Figure 5e). The appropriate mechanical properties of SPI‐Li‐based SPEs should be considered, which is very important for the construction of durable and dendritic‐free solid‐state LIB. Incorporation of LiTFSI greatly affected the mechanical properties of the SPI‐Li based SPEs, evidenced by the decreased tensile strength of SPI‐Li based SPEs (Figure 5f, Figure S14, Supporting Information). However, the ductility of SPI‐3Li was greatly improved, achieving a tensile strength of 5 MPa, which is much higher than that of traditional PEO electrolyte membranes (0.55 MPa).^[^ ^36^ ^]^ It is also worth noting that SPI‐3Li has higher strength and ionic conductivity than traditional PEO‐based polymer electrolytes (Figure 5g).^[^ ^9^, ^36^, ^38^, ^39^, ^40^, ^41^, ^42^, ^43^, ^44^ ^]^

## 3 Conclusion

In summary, we demonstrated the flexible SPEs based on the dynamic network of SPI imine bonds. The SPI‐based vitrimer with LiTFSI as an ionic conductive filler presented high mechanical strength, reproducible, and non‐flammable. This inexpensive and catalyst‐free SPI‐based vitrimer matrix exhibited an Arrhenius‐like ductility to heat and can be recycled and reused for multiple generations. The malleable vitrimer matrix of imine covalent cross‐linked network can also be recycled and reshaped using water at ambient temperature, which may realize the green processing of materials. Benefiting from the interaction between SPI and TFSI anion, numerous free Li^+^ improve the transport capacity of Li^+^ in the electrolyte. Consequently, the SPEs exhibited an excellent ionic conductivity of 3.3 × 10^−4^ S cm^‐1^ at 30 °C and thermal stability up to 240 °C. Thus, the SPEs based on the imine bond dynamic network of SPI is a promising platform for recyclable and repairable electrolytes.

## Conflict of Interest

The authors declare no conflict of interest.

## Supporting information

Supporting Information

## Data Availability

The data that support the findings of this study are available from the corresponding author upon reasonable request.

## References

[1] a) H. Li , D. Wu , J. Wu , L. Y. Dong , Y. J. Zhu , X. Hu , Adv. Mater. 2017, 29, 1703548; 10.1002/adma.201703548 29044775

[2] C. F. J. Francis , I. L. Kyratzis , A. S. Best , Adv. Mater. 2020, 32, e1904205. 10.1002/adma.201904205 31957144

[3] A. Manthiram , X. Yu , S. Wang , Nat. Rev. Mater. 2017, 2, 16103.

[4] a) F. Wu , N. Chen , R. Chen , Q. Zhu , J. Qian , L. Li , Chem. Mater. 2016, 28, 848;

[5] a) J. Bae , Y. Li , J. Zhang , X. Zhou , F. Zhao , Y. Shi , J. B. Goodenough , G. Yu , Angew. Chem., Int. Ed. Engl. 2018, 57, 2096; 29314472 10.1002/anie.201710841

[6] a) Z. Fan , B. Ding , T. Zhang , Q. Lin , V. Malgras , J. Wang , H. Dou , X. Zhang , Y. Yamauchi , Small 2019, 15, e1903952; 31565864 10.1002/smll.201903952

[7] a) Q. Zhao , X. Liu , S. Stalin , K. Khan , L. A. Archer , Nat. Energy 2019, 4, 365;

[8] J. Wan , J. Xie , X. Kong , Z. Liu , K. Liu , F. Shi , A. Pei , H. Chen , W. Chen , J. Chen , X. Zhang , L. Zong , J. Wang , L. Q. Chen , J. Qin , Y. Cui , Nat. Nanotechnol. 2019, 14, 705. 31133663 10.1038/s41565-019-0465-3

[9] W. Tang , S. Tang , C. Zhang , Q. Ma , Q. Xiang , Y. W. Yang , J. Luo , Adv. Energy Mater. 2018, 8, 1800866.

[10] a) J. Zhang , J. Zhao , L. Yue , Q. Wang , J. Chai , Z. Liu , X. Zhou , H. Li , Y. Guo , G. Cui , L. Chen , Adv. Energy Mater. 2015, 5, 1501082;

[11] a) Q. Lu , Y.‐B. He , Q. Yu , B. Li , Y. V. Kaneti , Y. Yao , F. Kang , Q.‐H. Yang , Adv. Mater. 2017, 29, 1604460; 10.1002/adma.201604460 28145599

[12] B. B. Jing , C. M. Evans , J. Am. Chem. Soc. 2019, 141, 18932. 31743006 10.1021/jacs.9b09811

[13] J. Deng , X. Kuang , R. Liu , W. Ding , A. C. Wang , Y. C. Lai , K. Dong , Z. Wen , Y. Wang , L. Wang , H. J. Qi , T. Zhang , Z. L. Wang , Adv. Mater. 2018, 30, e1705918. 10.1002/adma.201705918 29457281

[14] a) W. Denissen , J. M. Winne , F. E. Du Prez , Chem. Sci. 2016, 7, 30; 28757995 10.1039/c5sc02223a PMC5508697

[15] a) M. Chen , L. Zhou , Y. Wu , X. Zhao , Y. Zhang , ACS Macro Lett. 2019, 8, 255; 10.1021/acsmacrolett.9b00015 35650825

[16] a) D. Montarnal , M. Capelot , F. Tournilhac , L. Leibler , Science 2011, 334, 965; 22096195 10.1126/science.1212648

[17] M. Delahaye , J. M. Winne , F. E. Du Prez , J. Am. Chem. Soc. 2019, 141, 15277. 31469270 10.1021/jacs.9b07269

[18] a) A. P. Bapat , B. S. Sumerlin , A. Sutti , Mater. Horiz. 2020, 7, 694;

[19] P. Taynton , H. Ni , C. Zhu , K. Yu , S. Loob , Y. Jin , H. J. Qi , W. Zhang , Adv. Mater. 2016, 28, 2904. 26875745 10.1002/adma.201505245

[20] H. Liu , H. Zhang , H. Wang , X. Huang , G. Huang , J. Wu , Chem. Eng. J. 2019, 368, 61.

[21] a) D. J. Fortman , R. L. Snyder , D. T. Sheppard , W. R. Dichtel , ACS Macro Lett. 2018, 7, 1226; 10.1021/acsmacrolett.8b00667 35651259

[22] a) Y. Zhuo , Z. Xia , Y. Qi , T. Sumigawa , J. Wu , P. Sestak , Y. Lu , V. Hakonsen , T. Li , F. Wang , W. Chen , S. Xiao , R. Long , T. Kitamura , L. Li , J. He , Z. Zhang , Adv. Mater. 2021, 33, e2008523; 10.1002/adma.202008523 PMC11468028 33938044

[23] Q. Guo , X. Zhang , F. Zhao , Q. Song , G. Su , Y. Tan , Q. Tao , T. Zhou , Y. Yu , Z. Zhou , C. Lu , ACS Nano 2020, 14, 2788. 32045216 10.1021/acsnano.9b09802

[24] a) J. Cao , C. Lu , J. Zhuang , M. Liu , X. Zhang , Y. Yu , Q. Tao , Angew. Chem., Int. Ed. Engl. 2017, 56, 8795; 28544097 10.1002/anie.201704217

[25] P. Taynton , K. Yu , R. K. Shoemaker , Y. Jin , H. J. Qi , W. Zhang , Adv. Mater. 2014, 26, 3938. 24677455 10.1002/adma.201400317

[26] M. Capelot , D. Montarnal , F. Tournilhac , L. Leibler , J. Am. Chem. Soc. 2012, 134, 7664. 22537278 10.1021/ja302894k

[27] X. Fu , L. Scudiero , W.‐H. Zhong , J. Mater. Chem. A 2019, 7, 1835.

[28] D. D. Ordinario , L. Phan , W. G. t. Walkup , J. M. Jocson , E. Karshalev , N. Husken , A. A. Gorodetsky , Nat. Chem. 2014, 6, 596. 24950329 10.1038/nchem.1960

[29] a) X. Fu , Y. Jewel , Y. Wang , J. Liu , W. H. Zhong , J. Phys. Chem. Lett. 2016, 7, 4304; 27740773 10.1021/acs.jpclett.6b02071

[30] a) C. Wang , H. Zhang , S. Dong , Z. Hu , R. Hu , Z. Guo , T. Wang , G. Cui , L. Chen , Chem. Mater. 2020, 32, 9167;

[31] J. Xiang , Y. Zhang , B. Zhang , L. Yuan , X. Liu , Z. Cheng , Y. Yang , X. Zhang , Z. Li , Y. Shen , J. Jiang , Y. Huang , Energy Environ. Sci. 2021, 14, 3510.

[32] R. Rojaee , S. Cavallo , S. Mogurampelly , B. K. Wheatle , V. Yurkiv , R. Deivanayagam , T. Foroozan , M. G. Rasul , S. Sharifi‐Asl , A. H. Phakatkar , M. Cheng , S. B. Son , Y. Pan , F. Mashayek , V. Ganesan , R. Shahbazian‐Yassar , Adv. Funct. Mater. 2020, 30, 1910749.

[33] a) K. Kimura , J. Motomatsu , Y. Tominaga , J. Phys. Chem. C 2016, 120, 12385;

[34] a) Y. Yamada , M. Yaegashi , T. Abe , A. Yamada , Chem. Commun. 2013, 49, 11194; 10.1039/c3cc46665e 24150285

[35] a) N. A. Stolwijk , C. Heddier , M. Reschke , M. Wiencierz , J. Bokeloh , G. Wilde , Macromolecules 2013, 46, 8580;

[36] K. He , S. H. Cheng , J. Hu , Y. Zhang , H. Yang , Y. Liu , W. Liao , D. Chen , C. Liao , X. Cheng , Z. Lu , J. He , J. Tang , R. K. Y. Li , C. Liu , Angew. Chem., Int. Ed. Engl. 2021, 60, 12116. 33723915 10.1002/anie.202103403

[37] Y. Cui , J. Wan , Y. Ye , K. Liu , L. Y. Chou , Y. Cui , Nano Lett. 2020, 20, 7. 10.1021/acs.nanolett.9b04815 32020809

[38] R. Bouchet , S. Maria , R. Meziane , A. Aboulaich , L. Lienafa , J.‐P. Bonnet , T. N. Phan , D. Bertin , D. Gigmes , D. Devaux , Nat. Mater. 2013, 12, 452. 23542871 10.1038/nmat3602

[39] Y. Lin , X. Wang , J. Liu , J. D. Miller , Nano Energy 2017, 31, 478.

[40] H. Wang , Q. Wang , X. Cao , Y. He , K. Wu , J. Yang , H. Zhou , W. Liu , X. Sun , Adv. Mater. 2020, 32, e2001259. 10.1002/adma.202001259 32734684

[41] D. G. Mackanic , W. Michaels , M. Lee , D. Feng , J. Lopez , J. Qin , Y. Cui , Z. Bao , Adv. Energy Mater. 2018, 8, 1800703.

[42] Y. Li , Z. Sun , D. Liu , S. Lu , F. Li , G. Gao , M. Zhu , M. Li , Y. Zhang , H. Bu , Z. Jia , S. Ding , Energy Environ. Mater. 2020, 4, 434.

[43] J. Hu , P. He , B. Zhang , B. Wang , L.‐Z. Fan , Energy Storage Mater. 2020, 26, 283.

[44] D. G. Mackanic , X. Yan , Q. Zhang , N. Matsuhisa , Z. Yu , Y. Jiang , T. Manika , J. Lopez , H. Yan , K. Liu , X. Chen , Y. Cui , Z. Bao , Nat. Commun. 2019, 10, 5384. 31772158 10.1038/s41467-019-13362-4 PMC6879760

[45] Z. Huang , Q. Pan , D. M. Smith , C. Y. Li , Adv. Mater. Interfaces 2018, 6, 1801445.

