# Supplementary material for: Recyclable, Self‐Healing Solid Polymer Electrolytes by Soy Protein‐Based Dynamic Network
Source: Adv Sci (Weinh). 2022 Feb 10;9(11):2103623. doi: 10.1002/advs.202103623 (PMC9008422; doi:10.1002/advs.202103623)
Supplement: Supplementary file 1 — Supporting Information [file ADVS-9-2103623-s001.pdf]

ADVANCED SCIENCE
Open Access

Supporting Information

for *Adv. Sci.*, DOI 10.1002/advs.202103623

Recyclable, Self-Healing Solid Polymer Electrolytes by Soy Protein-Based Dynamic Network

Weidong Gu, Feng Li, Tao Liu, Shanshan Gong, Qiang Gao, Jianzhang Li and Zhen Fang**

ADVANCED SCIENCE
Open Access

Supporting Information

for *Adv. Sci.,* DOI: 10.1002/advs.202103623

Recyclable, Self-healing Solid Polymer Electrolytes by Soy Protein-based Dynamic Network

Weidong Gu [a], Feng Li [a], Tao Liu [a], Shanshan Gong [a], Qiang Gao [a], Jianzhang Li [a], Zhen Fang [b]**

Supporting Information

Recyclable, Self-healing Solid Polymer Electrolytes by Soy Protein-based Dynamic Network

Weidong Gu, [a, b] Feng Li, [a, b] Tao Liu, [a, b] Shanshan Gong, [a, b] Qiang Gao [a, b] Jianzhang Li [a, b]*

[a] Beijing Advanced Innovation Center for Tree Breeding by Molecular Design (Beijing Forestry University), Beijing 100083, China

[b] Beijing Key Laboratory of Wood Science and Engineering & MOE Key Laboratory of Wooden Material Science and Application, Beijing Forestry University, Beijing 100083, China

*Corresponding author. lijzh@bjfu.edu.cn (J. Li).

Materials

Soy protein isolate (SPI,95% protein) was provided by Yuwang Ecological Food Industry Co., Ltd. Adipic acid (purity ≥ 98%), diethylenetriamine (purity ≥ 99%), p-toluenesulfonic acid (purity ≥ 99%), 1,4-Phthalaldehyde (PA) (purity ≥ 99%) and 1,4-Butanediol diglycidyl ether (BDE) (purity ≥ 95%), bis(trifluoromethane) sulfonimide lithium (LiTFSI) (purity ≥ 99%) were purchased from Shanghai McLean Biochemical Technology Co., Ltd.

Synthesis of hyperbranched polyamide (HBPA)

First, adipic acid (146.14 g, 1 mol) and diethylenetriamine (154.75 g, 1.5 mol) were mixed in a three-necked glass reactor flask at 80 °C under constant stirring. Second, *p*-toluenesulfonic acid (2.58 g, 0.015 mol) was added to the flask, and the resulting solution was maintained at 160 °C for 5 h. then cooled to 120 ℃. Then, 150 g of hot water (80 ℃) was slowly added following with the addition of another 150 g of distilled water after five minutes. The mixture was stirred at 70 ℃ for half an hour, reduced the temperature to 30 ℃ to yield HBPA with a concentration of about 50%.

Preparation of SPI-based vitrimer films

SPI (5 g) was added to 95 mL of distilled water and the resulting solution was stirred vigorously for 10 min at room temperature. The pH was then adjusted to 10 with a NaOH solution (10 wt%) before heating the mixture at 85 °C for 30 min with magnetically stirring. A certain amount of HBPA, PA and BDE (as listed in Table S1) was dispersed in the SPI solution and stirring continued under 75 ℃ for 3 h. The mixtures were poured into a Teflon-coated dish, then dried in an oven at 45 °C for 48 h to remove the water. The obtained films showed excellent mechanical properties when the optimized weight ratio of SPI:HBPA was 1:1 and the optimized molar ratio of PA:BDE was 1:1. To preparation of SPI-based solid polymer electrolytes, different amounts of LiTFSI was then added to the above homogeneous solution and stirred for 1 h. The forming and drying procedures were identical to that used for SPI-based vitrimer films.

Characterization

Fourier transform infrared (FTIR) spectra of the SPI-based films were recorded using a Nicolet 6700 infrared spectrophotometer (in the range from 500 to 4000 cm^{-1} with a resolution of 4 cm^{-1} at ambient temperature). This spectrometer was also used for the temperature dependent FTIR experiments from 30 to 120 °C with a heating rate of 3 °C/min. The atomic percentage of N was determined by Xray photoelectron spectroscopy (XPS) measurements, which were performed on a Thermo Scientific ESCALAB Xi+. The mechanical properties of the SPI-based films were determined using an Instron 3366 instrument. Electrochemical impedance spectroscopy (EIS) measurements were taken using an Agilent E4990A with a 1 V amplitude between 1 MHz and 20 Hz. Differential scanning calorimetry (DSC) measurements were conducted using a TA Q2000 under a N_2 atmosphere over a temperature range of -70-180 °C at a heating rate of 10 °C/ min. Thermogravimetric analysis (TGA) was performed on a TA Q50 over a temperature range of 25-600 °C and a heating rate of 10 °C min^{-1} under a N_2 atmosphere. The relaxation moduli measured by dynamic mechanical analysis (DMA) is the dynamic modulus, and the Young's modulus calculated from the slope of the stress–strain curve should be the static tensile modulus. For DMA by TA Q800, the sample was heated from -20 to 180 °C at a heating rate of 3 K/min at a tensile mode of 0.1% dynamic strain, and the frequency was set at 1 Hz. According to the ASTM D882-12 standard, the Instron 3366 instrument was used to determine the mechanical properties of the SPI-based vitrimer films at a loading speed of 20 mm min^{-1} and an initial gauge length of 50 mm. The specimen is a rectangle of 100×10 mm, and the test is repeated at least 6 times for each type of film. Raman spectra were recorded using a HR-800 (Horiba), by averaging 2 scans per sample over the range 400-1000 cm^{-1} with the resolution of 1 cm^{-1}.For stress relaxation test, the sample was preloaded with a force of 10^{-3} N and stretched to a constant strain of 1% at the required temperature (55-100 ℃) for 5 minutes, and then the stress attenuation was monitored over time. In this study, the electrochemical tests were carried out using CR2025-type coin cells. The SPI-3Li film was used as SPEs, $LiFePO_4$ was used as the positive electrode, and lithium was used

as the negative electrode.

Statistics

The experiment data of mechanical properties were presented as the mean standard deviation (SD) of 6 (n) samples. P-values are calculated using one-way analysis of variance (ANOVA) and the differences were statically significant at $p < 0.001$. Data were analyzed by using the SPSS software.

The relationship between the relaxation time τ and the temperature is fitted by Arrhenius' law following the formula below:

$$\tau = \tau_0 \exp\left(\frac{E_a}{RT}\right) \qquad (S1)$$

where τ is the relaxation time at temperature of *T*, *T* is the absolute temperature, τ_0 is the relaxation time at a reference temperature, E_a is a stress relaxation activation energy, and the *R* is the gas constant.

The rate performance and cycle performance of coin half-cell were tested at 60 ℃. The D_{Li+} value can be calculated according to the following formula (S2):

$$D = \frac{4}{\pi\tau}\left(\frac{n_m V_m}{S}\right)^2\left(\frac{\Delta E_S}{\Delta E_\tau}\right)^2 \qquad (S2)$$

τ is relaxation time; n_m is the moles number of the electrode material; V_m is the molar volume of the electrode material; S is the electrode/electrolyte contact area; ΔE_S is the voltage change caused by the pulse; ΔE_τ is the voltage change of constant current charge (discharge).

Narrow-scan XPS spectra of N 1s orbital (**Figure 1e, Figure S5**) and Raman spectra (**Figure 3d**) were fitted by Avantage and Origin2021b software, respectively.

Table S1. Formulations of the SPI-based films

Sample	SPI（g）	HBPA（g）	PA（g）	BDE（g）	Water（g）	LiTFSI（g）
**SPI_1**	1	1.5	0.388	0.583	19	
**SPI_2**	1	1	0.388	0.583	19	-
**SPI_3**	1	0.67	0.204	0.304	19	-
**$SPI_{3:2}$**	1	1	0.216	0.492	19	-
**$SPI_{1:1}$(SPI_2, SPI-0Li)**	1	1	0.280	0.416	19	-
**$SPI_{2:3}$**	1	1	0.326	0.328	19	-
**SPI-1Li**	1	1	0.280	0.416	19	1
**SPI-2Li**	1	1	0.280	0.416	19	2
**SPI-3Li**	1	1	0.280	0.416	19	3
**SPI-4Li**	1	1	0.280	0.416	19	4

Table S2. The 18 different amino acids in the Glycinin (11S globulin) and conglycinin (7S globulin).

Amino acid	Conglycinin (7S globulin)	Glycinin (11S globulin)	Amino acid	Conglycinin (7S globulin)	Glycinin (11S globulin)
**Glutamic acid**	24.17	25.11	**Isoleucine**	5.81	5.53
**Arginine**	9.70	8.75	**Tyrosine**	3.73	4.13
**Phenylalanine**	6.69	6.13	**Methionine**	0.87	1.51
**Valine**	5.29	5.85	**Leucine**	8.87	8.40
**Glycine**	3.55	4.96	**Lysine**	7.30	6.97
**Tryptophan**	0.90	1.36	**Alanine**	3.84	4.27
**Aspartic acid**	13.42	13.72	**Histidine**	2.69	2.53
**Threonine**	2.77	4.15	**Cystine**	0.52	1.22
**Serine**	5.77	6.17	**Proline**	5.38	6.21

Glycinin (11S globulin) and conglycinin (7S globulin) are two important components of soy protein, accounting for more than 80% of the total protein.[1]

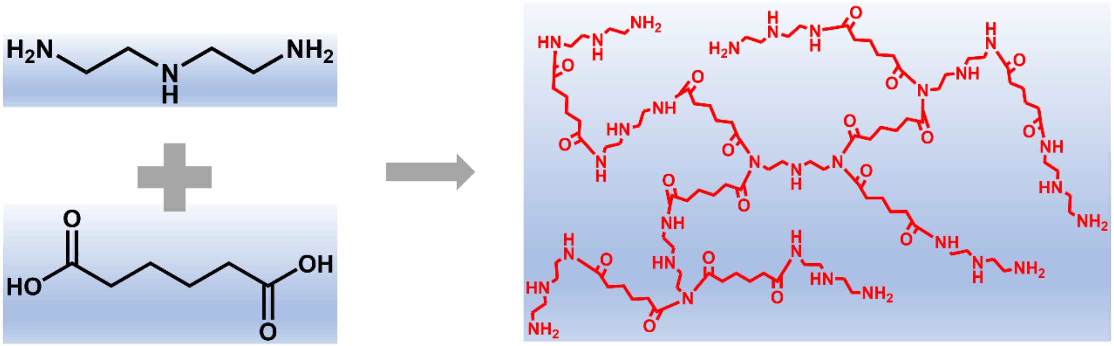

Figure S1. Synthesis of the hyperbranched polyamide (HBPA).

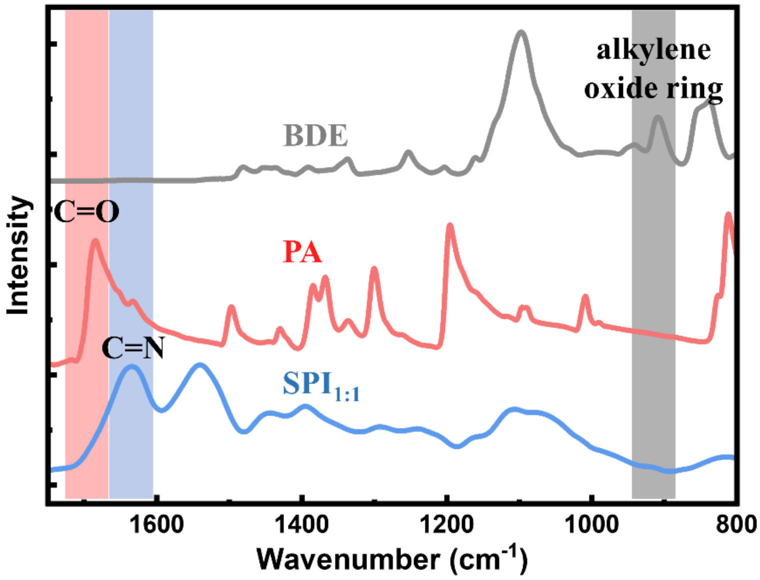

Figure S2. Fourier transform infrared (FTIR) spectrum of the 1,4-butanediol diglycidyl ether (BDE), 1,4-phthalaldehyde (PA), and $SPI_{1:1}$ based vitrimer.

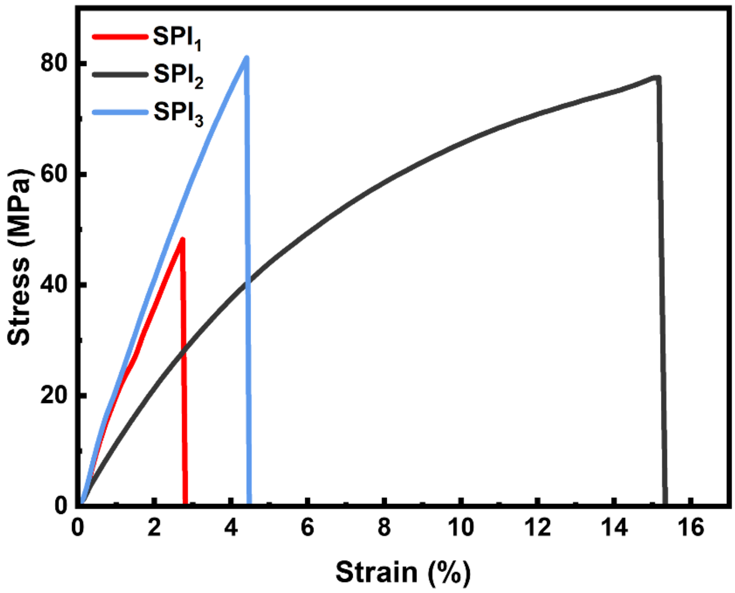

Figure S3. The stress-strain curve of SPI_1 (the mass ratio of SPI/HBPA is 3:2 and the molar ratio of PA/BDE is 1:1), SPI_2 (the mass ratio of SPI/HBPA is 1:1 and the molar ratio of PA/BDE is 1:1), and SPI_3 (the mass ratio of SPI/HBPA is 2:3 and the molar ratio of PA/BDE is 1:1) vitrimer films.

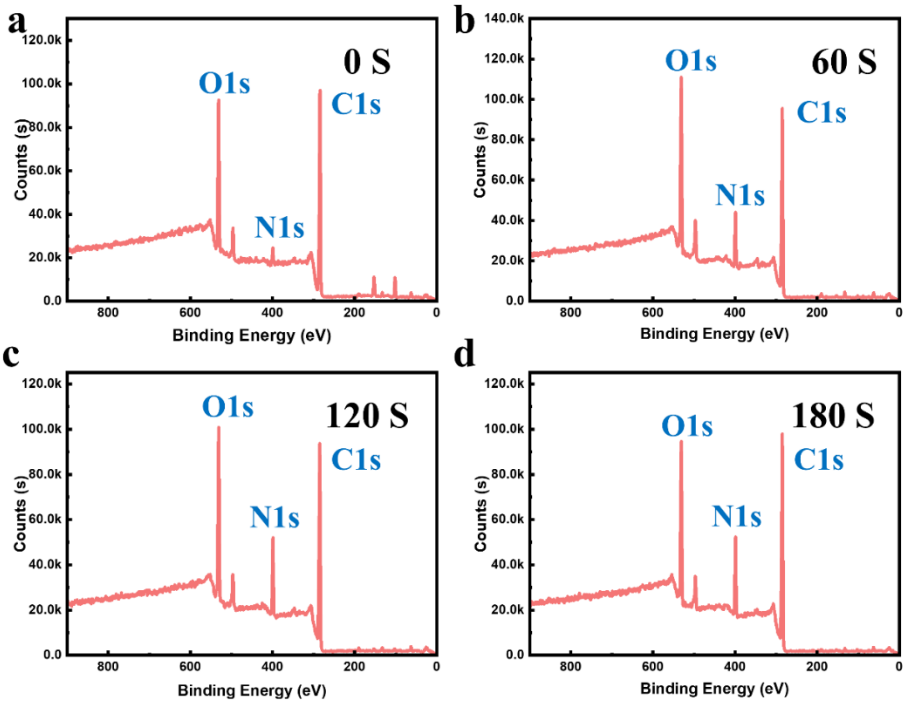

Figure S4. The wide scan XPS of the $SPI_{1:1}$ film under different time (the etching time is proportional to etching depth on the sample surface).

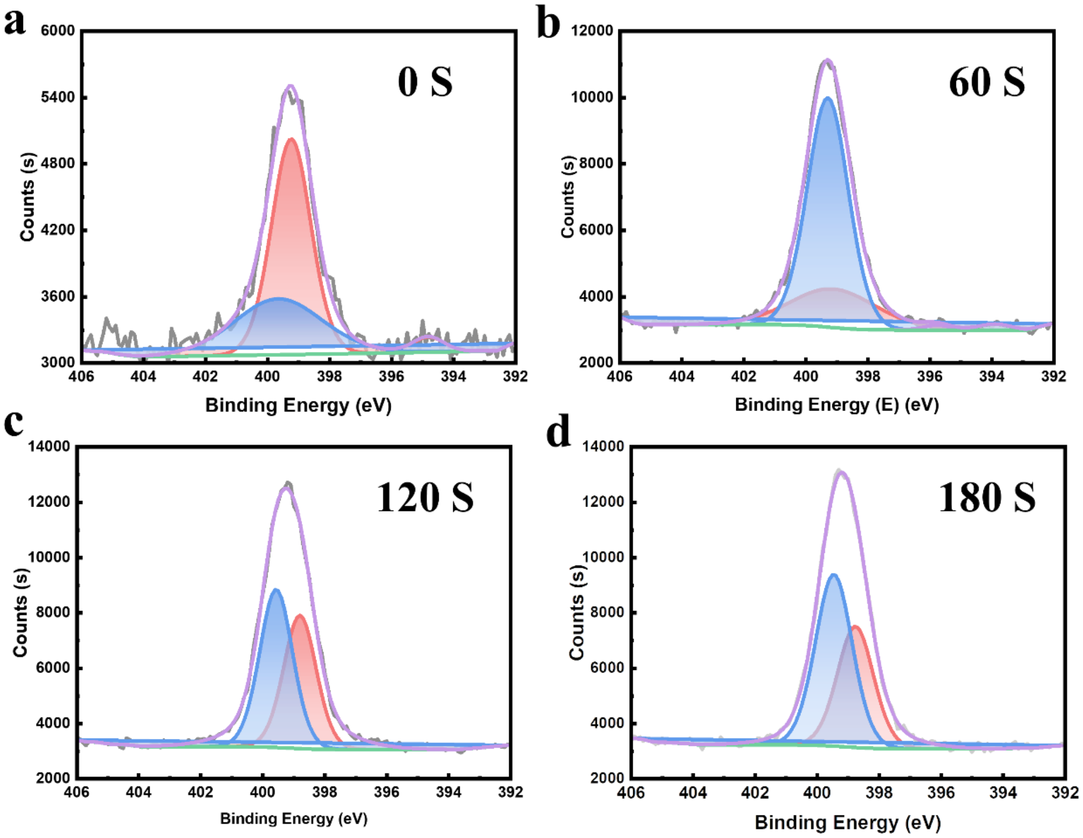

Figure S5. Narrow-scan XPS spectra of N 1s orbital and their fitting curves indicate the presence of both bonded N-H (red) and free N-H (blue) groups.

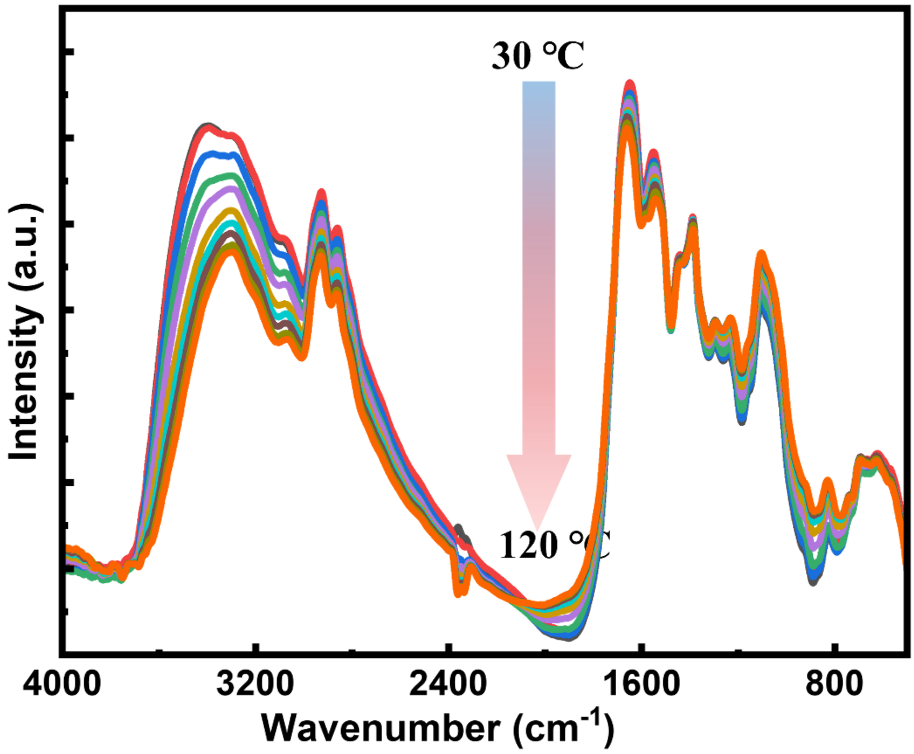

Figure S6. Temperature-dependent FTIR spectra (500 cm^{-1} to 4000 cm^{-1}) of $SPI_{1:1}$ upon heating from 30 to 120 °C

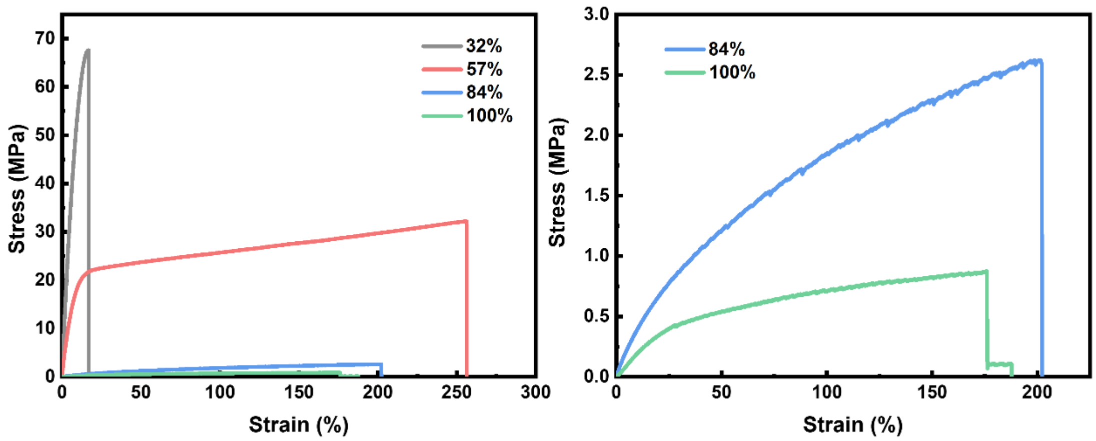

Figure S7. The stress-strain curve of $SPI_{1:1}$ film under different humidity. The $SPI_{1:1}$ film was placed in a container having a relative humidity (RH) of 32% (saturated magnesium chloride solution), 57% (saturated sodium bromide solution), 84% (saturated potassium chloride solution), and 100% (water) at 25 °C, respectively.

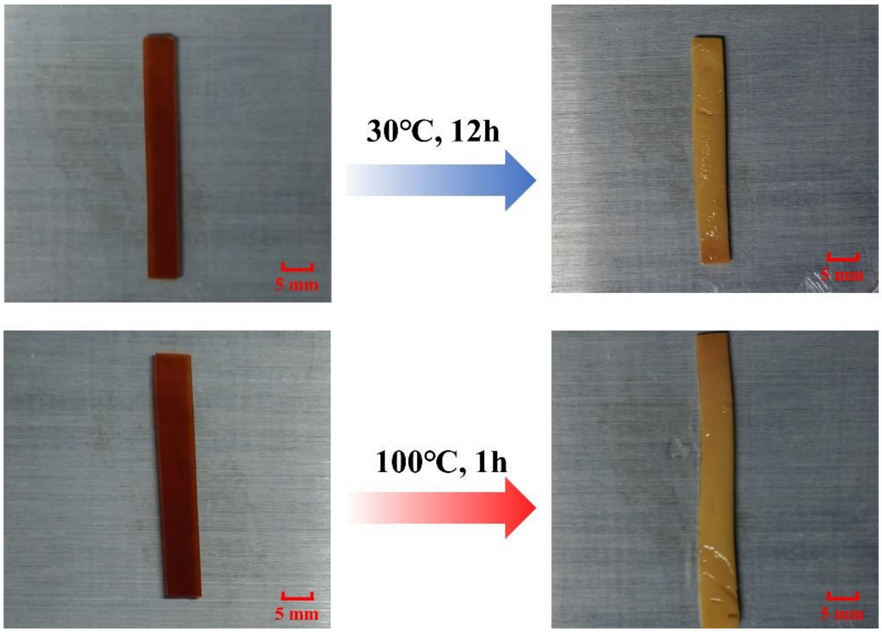

Figure S8. The photo of the $SPI_{1:1}$ vitrimer film taken from warm water (30°C, 12h) and boiling water (100°C, 1h).

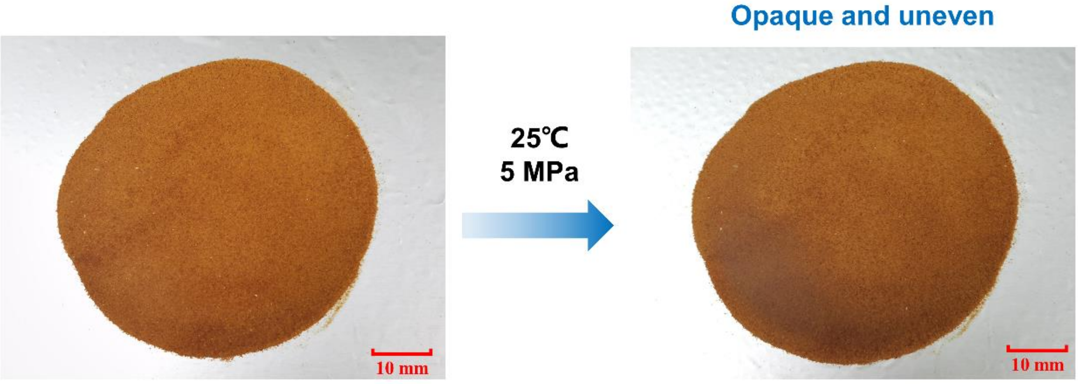

Figure S9. The $SPI_{1:1}$ vitrimer powder was cold-pressed under 25°C and 5 MPa for 24 h.

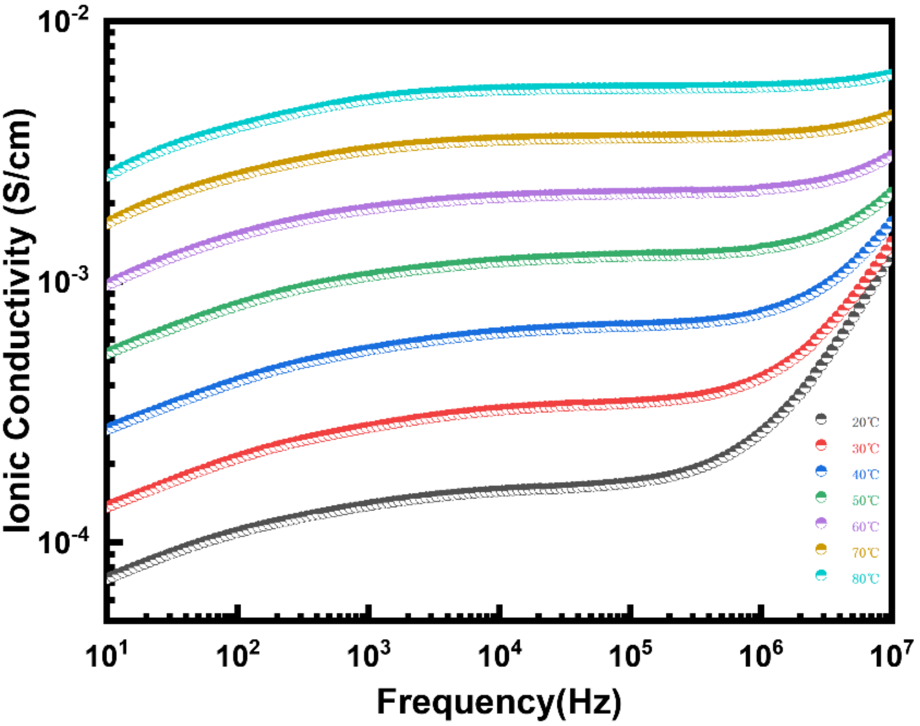

Figure S10. The ionic conductivity of the SPI-3Li film under different temperature with changing frequency.

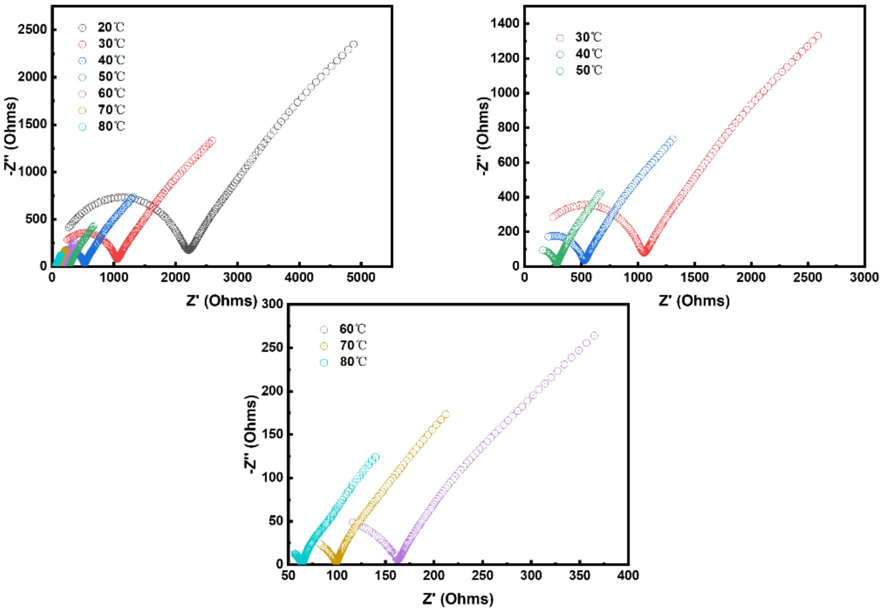

Figure S11. The EIS profiles of SPI-3Li electrolyte film at different temperature.

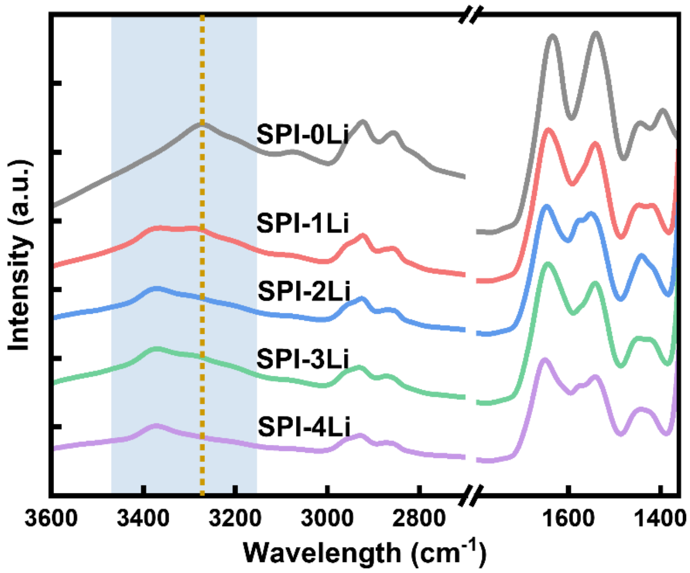

Figure S12. The FTIR of SPI-Li based SPEs with different proportions of LiTFSI, in the wavenumber range of 1400 cm^{-1} to 3600 cm^{-1}.

4, 1.

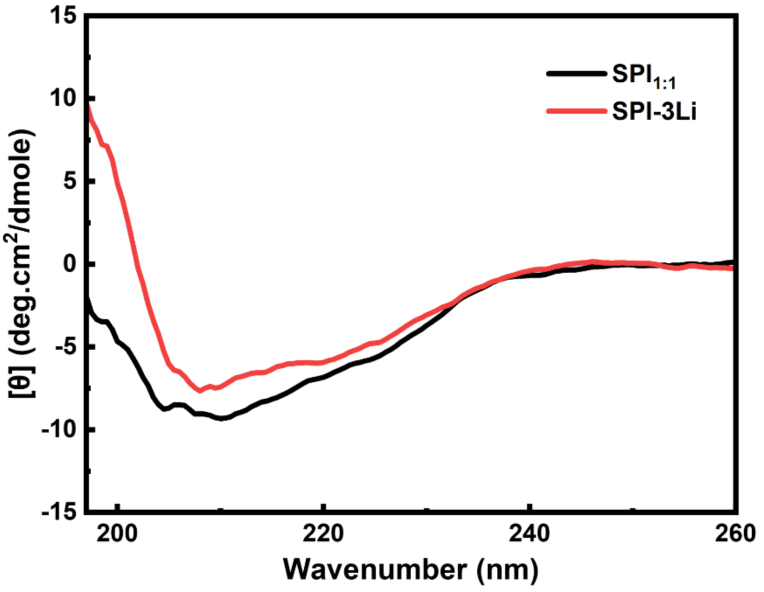

Figure S 13. Circular dichroism spectra of SPI1:1 and SPI-3Li.

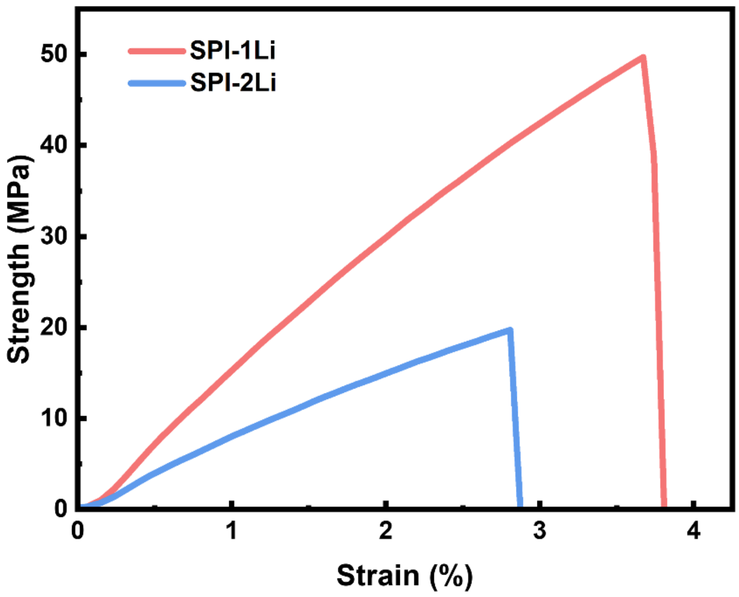

Figure S14. The stress-strain curve of SPI-1Li and SPI-2Li.

References

[1] M. K. Thakur, V. K. Thakur, R. K. Gupta, A. Pappu, ACS Sustainable Chem. Eng. 2015, 4, 1.
